# Factors Contributing to Congenital Syphilis Cases — New York City, 2010–2016

**DOI:** 10.15585/mmwr.mm6739a3

**Published:** 2018-10-05

**Authors:** Jennifer Sanderson Slutsker, Robin R. Hennessy, Julia A. Schillinger

**Affiliations:** ^1^Bureau of Sexually Transmitted Infections, New York City Department of Health and Mental Hygiene; ^2^Division of STD Prevention, National Center for HIV/AIDS, Viral Hepatitis, STD, and TB Prevention, CDC.

Congenital syphilis occurs when syphilis is transmitted from a pregnant woman to her fetus; congenital syphilis can be prevented through screening and treatment during pregnancy. Transmission to the fetus can occur at any stage of maternal infection, but is more likely during primary and secondary syphilis, with rates of transmission up to 100% at these stages (*1*). Untreated syphilis during pregnancy can cause spontaneous abortion, stillbirth, and early infant death. During 2013–2017, national rates of congenital syphilis increased from 9.2 to 23.3 cases per 100,000 live births (*2*), coinciding with increasing rates of primary and secondary syphilis among women of reproductive age (*3*). In New York City (NYC), cases of primary and secondary syphilis among women aged 15–44 years increased 147% during 2015–2016. To evaluate measures to prevent congenital syphilis, the NYC Department of Health and Mental Hygiene (DOHMH) reviewed data for congenital syphilis cases reported during 2010–2016 and identified patient-, provider-, and systems-level factors that contributed to these cases. During this period, 578 syphilis cases among pregnant women aged 15–44 years were reported to DOHMH; a congenital syphilis case was averted or otherwise failed to occur in 510 (88.2%) of these pregnancies, and in 68, a case of congenital syphilis occurred (eight cases per 100,000 live births).* Among the 68 pregnant women associated with these congenital syphilis cases, 21 (30.9%) did not receive timely (≥45 days before delivery) prenatal care. Among the 47 pregnant women who did access timely prenatal care, four (8.5%) did not receive an initial syphilis test until <45 days before delivery, and 22 (46.8%) acquired syphilis after an initial nonreactive syphilis test. These findings support recommendations that health care providers screen all pregnant women for syphilis at the first prenatal care visit and then rescreen women at risk in the early third trimester.

The 2009 U.S. Preventive Services Task Force (USPSTF) Recommendation Statement^†^ and 2015 CDC Sexually Transmitted Disease Treatment Guidelines recommend serologic syphilis screening for all women at first prenatal care visit and additional testing at 28–32 weeks’ gestation and at delivery for women at high risk (*4*). Whereas the USPSTF outlines specific groups which might be considered at high risk and recommended for testing during third trimester and at delivery (i.e., uninsured women, women living in poverty, sex workers, illicit drug users, women diagnosed with another sexually transmitted disease, and other women residing in communities with high syphilis morbidity), CDC recommends additional screening for “communities and populations in which the prevalence of syphilis is high and for women at high risk for infection” (*4*). New York State mandates syphilis screening at the first prenatal care examination^§^ and at delivery (*5*) and recommends repeat testing throughout pregnancy for women at high risk.^¶^ In NYC, the Health Code requires electronic reporting of reactive syphilis tests, as well as an indicator of pregnancy (known or probable). Women with reactive syphilis serologic tests who are known or suspected to be pregnant are the highest priority for investigation and are monitored throughout pregnancy.

DOHMH reviewed records of all pregnant women with reported syphilis (any stage) during 2010–2016, and all congenital syphilis cases that met surveillance case definitions for confirmed congenital syphilis, probable congenital syphilis, or syphilitic stillbirth.** The probable congenital syphilis definition includes infants with clinical findings suggesting congenital syphilis (infant criteria), infants born to women who received a diagnosis of syphilis during pregnancy and did not initiate penicillin-based treatment ≥30 days before delivery (maternal criteria), or both. Data on patients with congenital syphilis and their mothers were abstracted from DOHMH’s surveillance and case management registry and reviewed to determine whether prenatal care, syphilis screening, and treatment occurred early enough to prevent congenital syphilis. Both prenatal care and testing were defined as timely if received ≥45 days before delivery, the assumption being that 15 days is sufficient time for providers and DOHMH to follow up on reactive serology results and ensure treatment initiation ≥30 days before delivery, thereby preventing a probable congenital syphilis case.

During 2010–2016, a total of 578 syphilis infections were reported among women aged 15–44 years who were noted to be pregnant: six (1.0%) primary, 15 (2.6%) secondary, 126 (21.8%) early nonprimary nonsecondary, and 431 (74.6%) unknown duration or late. A total of 510 syphilis infections (88.2%) were not known to result in a congenital syphilis case. During this period, 68 congenital syphilis cases were reported. A median of eight cases were reported per year, with an increase to 19 cases in 2014 that was not sustained. Half of the 68 women who delivered an infant with congenital syphilis were aged 20–29 years, 53 (77.9%) were non-Hispanic black or Hispanic, and 31 of 56 (55.4%) with known country of origin were born outside the United States (Table 1).

**TABLE 1:** Demographic and clinical characteristics of mothers of infants with congenital syphilis cases (n = 68) — New York City, 2010–2016

Characteristic	No. (%)
**Age group (yrs)**	
15–19	5 (7.4)
20–29	34 (50.0)
30–39	24 (35.3)
40–49	5 (7.4)
**Race/Ethnicity**	
Black, non-Hispanic	29 (42.7)
Hispanic	24 (35.3)
White, non-Hispanic	5 (7.4)
Asian, non-Hispanic	3 (4.4)
Other	7 (10.3)
Area-based poverty level*	
Low (<10% below poverty)	6 (8.8)
Medium (10% to <20%)	18 (26.5)
High (20% to <30%)	17 (25.0)
Very high (≥30%)	27 (39.7)
**Country of birth^†^**	
Foreign-born	31 (55.4)
U.S.-born	25 (44.6)
**Syphilis stage^§^**	
Primary	2 (3.0)
Secondary	1 (1.5)
Early, non-primary, non-secondary	37 (56.1)
Unknown duration or late	26 (39.4)
**STIs reported before pregnancy^¶^**	
Syphilis only	11 (16.2)
Chlamydia only	9 (13.2)
Gonorrhea only	1 (1.5)
>1 previously reported STI	6 (8.8)
None	41 (60.3)
STIs reported during pregnancy**	
Chlamydia	6 (8.8)
None	62 (91.2)

**Abbreviation:** STI = sexually transmitted infection.

* Area-based poverty level categories are based on the percentage of the population in each zip code tabulation area with a household income below the poverty threshold set by the federal government. In alignment with local area-based poverty guidelines, five-year American Community Survey poverty data from 2011 to 2015 were used to divide zip code tabulation areas into four categories indicating the percentage of residents living below the federal poverty limit: low (<10 %), medium (10 to <20%), high (20% to <30%), and very high (≥30%). Pregnant women were assigned to a zip code tabulation area based on zip code of residence at the time of reporting.

^†^ Calculation of the percent of pregnant women by country of birth excludes women for whom country of birth was unknown.

^§^ Calculation of the percentage of pregnant women by syphilis stage excludes two pregnant women who did not meet the maternal criteria for reporting a congenital syphilis case. CDC case definitions were used to assign a syphilis stage to each pregnant woman (https://wwwn.cdc.gov/nndss/conditions/syphilis/case-definition/2018/).

^¶^ STIs reported before pregnancy include confirmed cases of syphilis (all stages), chlamydia, and gonorrhea reported to the New York City Department of Health and Mental Hygiene before each pregnant woman's estimated last menstrual period.

** STIs reported during pregnancy include confirmed cases of chlamydia reported to the New York City Department of Health and Mental Hygiene between each pregnant woman's estimated last menstrual period and delivery date. No pregnant woman in this investigation was reported with gonorrhea during this time.

Among these 68 mothers, 21 (30.9%) did not receive prenatal care or a syphilis test ≥45 days before delivery (Figure). Although DOHMH does not routinely record the reason why pregnant women with syphilis do not access prenatal care, 16 (76.2%) of 21 women had documented obstacles to accessing health care, such as substance use, mental health disorders, recent arrival in the United States, or unstable housing. During case investigation, five (23.8%) women cited lack of health care coverage as a reason for not seeking prenatal care.

**FIGURE:**
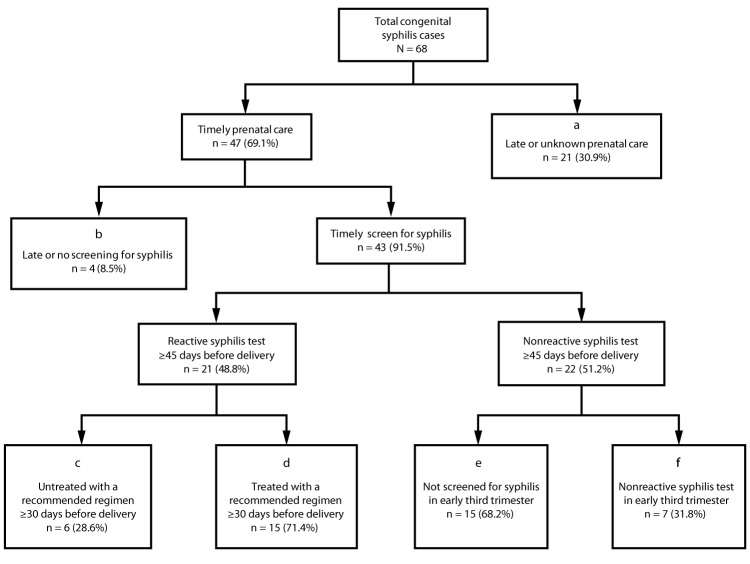
Clinical care and public health management of pregnancies among women who delivered an infant with congenital syphilis — New York City, 2010–2016*^,†,§^ * Box a includes pregnant women with no documentation of prenatal care or syphilis screening ≥45 days before delivery. Box b includes pregnant women with prenatal care documented ≥45 days before delivery but no documentation of syphilis screening ≥45 days before delivery. Box c includes pregnant women with documentation of a reactive test for syphilis ≥45 days before delivery and documentation of adequate treatment initiated <30 days before delivery or no documentation of adequate treatment initiated before delivery. Box e includes pregnant women with documentation of a nonreactive test for syphilis ≥45 days before delivery, no documentation of syphilis screening between 28 weeks’ gestation (estimated) and ≥45 days before delivery, and documentation of a reactive test <30 days before or at delivery such that infection was believed to have been acquired just before delivery. ^†^ Box d includes pregnant women who had a documented reactive test for syphilis, initiated adequate treatment ≥30 days before delivery, but nonetheless had changes in serologic tests indicating reinfection late in pregnancy (e.g., increased nontreponemal titers). Box f includes pregnant women with documentation of a nonreactive test for syphilis between 28 weeks’ gestation (estimated) and ≥45 days before delivery and documentation of a reactive test <30 days before or at delivery such that infection was believed to have been acquired just before delivery. ^§^ Box d includes two pregnant women who had stable nontreponemal titers during pregnancy (and therefore did not meet maternal criteria for reporting a congenital syphilis case), but who delivered an infant with signs and symptoms that met the infant criteria for a probable congenital syphilis case.

Four (5.9%) of the 68 women received timely prenatal care but were not tested for syphilis ≥45 days before delivery (Figure). Investigation revealed informatics errors as the reason two of these women were not screened (e.g., syphilis serologies were not included when programming a prenatal “lab order set” into a new laboratory ordering system). These errors occurred in different health systems. One of these women's infant died shortly after birth.

Among the 68 women, 22 (32.4%) had a time-appropriate, nonreactive test and subsequently acquired syphilis during pregnancy (Figure). Among these women, 15 (68.2%) did not have a documented syphilis test during the early third trimester (Figure), including 12 (80.0%) who had at least one characteristic indicating risk for syphilis: 10 lived in a high-morbidity neighborhood,^††^ 11 resided in a high-poverty neighborhood,^§§^ one received a diagnosis of chlamydia during pregnancy, and two had syphilis before pregnancy. One woman who had a nonreactive test in the second trimester was not screened again until delivery, despite being seen in an emergency department with syphilis symptoms during the third trimester; her infant was stillborn.

The remaining 21 (30.9%) women had a reactive syphilis test ≥45 days before delivery. Six (28.6%) of these women had inadequate maternal treatment (Figure) because treatment was initiated too late or not at all. For one woman with inadequate treatment, investigation was delayed because pregnancy status was not known to DOHMH; for another woman, a provider advised delaying treatment, and the woman was not treated until <30 days before delivery. The remaining 15 (71.4%) initiated treatment ≥30 days before delivery but had stable or increasing nontreponemal titers consistent with reinfection or persistent infection close to delivery (Figure).

Among the 68 congenital syphilis cases were one syphilitic stillbirth (1.5%) and another confirmed case (1.5%) in an infant who later died. The remaining 66 congenital syphilis cases were probable; two (3.0%) met only infant criteria, 19 (28.8%) met both infant and maternal criteria, and 45 (68.2%) met only maternal criteria (Table 2). Many of the 45 infants who met only maternal criteria lacked documentation of a thorough congenital syphilis examination, 25 (55.6%) lacked long-bone radiograph results, and 26 (57.8%) lacked cerebrospinal fluid white blood cell count and protein analysis findings.

**TABLE 2:** Case definition criteria* associated with 66^†^ reported probable congenital syphilis cases — New York City, 2010–2016

Characteristic	Maternal criteria only (N = 45)	Infant criteria only (N = 2)	Maternal and infant criteria (N = 19)
	No. (%)	No. (%)	No. (%)
**Physical sign**	0 (—)	0 (—)	1 (5.3)
**Long-bone radiograph**			
Changes consistent with CS	0 (—)	1 (50.0)	1 (5.3)
No signs of CS	20 (44.4)	1 (50.0)	15 (78.9)
Not done	20 (44.4)	0 (—)	3 (15.8)
Unknown	5 (11.1)	0 (—)	0 (—)
**CSF VDRL analysis**			
Reactive	0 (—)	0 (—)	2 (10.5)
Nonreactive	34 (75.6)	2 (100.0)	15 (78.9)
Not done	9 (20.0)	0 (—)	1 (5.3)
Unknown	2 (4.4)	0 (—)	1 (5.3)
**CSF WBC and protein**			
Either elevated	3 (6.7)	2 (100.0)	18 (94.7)
Neither elevated	16 (35.6)	0 (—)	1 (5.3)
Not done	16 (35.6)	0 (—)	0 (—)
Unknown	10 (22.2)	0 (—)	0 (—)

**Abbreviations:** CS = congenital syphilis; CSF = cerebrospinal fluid; VDRL = venereal disease research laboratory nontreponemal serologic syphilis test; WBC = white blood cell.

* The probable CS case definition includes infants with clinical findings suggesting CS (infant criteria), infants born to women who received a diagnosis of syphilis during pregnancy and did not initiate penicillin-based treatment ≥30 days before delivery (maternal criteria), or both. Clinical signs of CS included are the indicators outlined in the infant/child criteria for reporting a CS case (https://wwwn.cdc.gov/nndss/conditions/congenital-syphilis/).

^†^ One confirmed case of CS in an infant who later died and one syphilitic still birth are excluded from this table.

## Discussion

Approximately 88% of syphilis infections among NYC women noted to be pregnant during 2010–2016 did not result in congenital syphilis, presumably because of early screening and treatment, underscoring the critical role that provider and public health systems play in preventing congenital syphilis. Nevertheless, 68 congenital syphilis cases were reported during this period, and analysis of these cases provides insight into factors contributing to these preventable infections.

In approximately one third of congenital syphilis cases, the major contributing factor was late initiation of prenatal care; lack of health care coverage was often cited by patients as a barrier to seeking care. Citywide in 2015, 83.2% of new mothers initiated prenatal care during the first trimester,^¶¶^ reflecting the expanded health insurance options available to pregnant women in New York, regardless of immigration status, through Medicaid and the New York health insurance marketplace.*** Absent or late prenatal care among mothers of infants with congenital syphilis suggests that pregnant women with syphilis might be unaware of available services or face barriers to obtaining prenatal care; this might be particularly applicable for women born outside the United States.

CDC identified improvement of electronic medical records as an essential area for reversing increases in congenital syphilis.^†††^ This investigation found two women with timely prenatal care who were not screened for syphilis because of errors in electronic systems, one of whose pregnancy resulted in an infant death. These cases emphasize the importance of data system functionality, such as clinical decision support tools and automated ordering of prenatal laboratory test panels aimed at ensuring syphilis screening in early pregnancy.

Testing all pregnant women early in pregnancy and retesting women at high risk at 28–32 weeks’ gestation and at delivery is recommended by CDC (*4*) and the USPSTF. In this investigation, few mothers of infants with congenital syphilis who acquired syphilis after an initial nonreactive test were screened in the early third trimester, despite that most (80%) could be considered at increased risk for syphilis. This finding points to the need for local guidance and provider training regarding characteristics that indicate a high risk for infection and a need for third-trimester screening. To encourage early detection of syphilis in pregnant women, some states have mandated screening at the first prenatal care examination and during the early third trimester. Universal third-trimester screening effectively prevented most congenital syphilis cases in Florida and Louisiana (*6*); however, this strategy might not be cost-effective in low-morbidity areas (*7*).

Finally, only two cases met the definition for a confirmed case or syphilitic stillbirth. Among probable cases, most met the surveillance definition solely by maternal criteria and had minimal signs of disease. These cases highlight the challenges inherent in both defining and diagnosing congenital syphilis. The surveillance definition for congenital syphilis intentionally values sensitivity at the expense of specificity, with the goal of maximizing identification of infants potentially infected with syphilis, an important compromise given that the laboratory and radiologic tests required for diagnosis might not be collected, and infants might be asymptomatic at birth (*8*).

The findings in this report are subject to at least two limitations. First, data came from DOHMH’s surveillance registry, and some are missing or incomplete. Second, NYC has a relatively small number of congenital syphilis cases^§§§^ and a syphilis epidemic that is largely driven by men who have sex with men (*9*), and results might not be generalizable to other jurisdictions.

Although no sustained increase in congenital syphilis occurred in NYC during 2010–2016, analysis of 68 cases identified areas where prevention measures might be enhanced. Syphilis screening during pregnancy is critical to preventing congenital syphilis. Health care systems can support screening by ensuring that syphilis tests can be electronically ordered, tracked, received, and flagged for review when results are missing or reactive. In addition, clear guidance regarding third-trimester screening could help identify and treat pregnant women who acquire syphilis during pregnancy.

**Summary**

**What is already known about this topic?**

Cases of congenital syphilis are increasing in the United States and often represent missed opportunities for prevention.

**What is added by this report?**

During 2010–2016, 578 New York City women with syphilis infection were noted to be pregnant, and in 510 (88.2%) pregnancies congenital syphilis did not occur. In the majority of the 68 congenital syphilis cases, maternal syphilis diagnosis occurred too late to prevent congenital syphilis.

**What are the implications for public health practice?**

Provider and public health systems play a critical role in preventing congenital syphilis through screening and treating pregnant women for syphilis; these systems need to be maintained and strengthened.

## Footnotes

* The number of live births come from NYC Vital Statistics data. https://a816-healthpsi.nyc.gov/epiquery/Birth/index.html.

† The 2009 USPSTF recommendation statement (Grade A) recommended screening all pregnant women for syphilis at the first prenatal care visit. The recommendation included an additional consideration to test women at high risk for syphilis again during the third trimester and at delivery and specifies groups at increased risk as uninsured women, women living in poverty, sex workers, illicit drug users, women diagnosed with another sexually transmitted disease, and women residing in communities with high syphilis morbidity. https://www.uspreventiveservicestaskforce.org/Page/Document/ClinicalSummaryFinal/syphilis-infection-in-pregnancy-screening. The 2018 USPSTF reaffirmation statement recommends screening all pregnant women “when they first present to care” and includes a consideration to rescreen women at high risk for syphilis in early third trimester and at delivery. In the 2018 recommendation, women at high risk include “those living in communities or geographic areas with higher prevalence of syphilis, those living with HIV, and those with a history of incarceration or commercial sex work.” https://www.uspreventiveservicestaskforce.org/Page/Document/RecommendationStatementFinal/syphilis-infection-in-pregnancy-screening1.

§ New York Public Health Law Section 2308 mandates syphilis screening at the first prenatal care examination. https://www.nysenate.gov/legislation/laws/PBH/2308.

¶ New York State recommends repeat testing throughout pregnancy for women at high risk, including patients in “communities and populations with high syphilis prevalence or for patients at high risk.” https://www.health.ny.gov/diseases/communicable/syphilis/treatment_guidelines/guidelines.htm.

** During 2010–2016, cases of congenital syphilis were categorized in accordance with the Council of State and Territorial Epidemiologists case definitions. https://wwwn.cdc.gov/nndss/conditions/congenital-syphilis.

†† Pregnant women were assigned to one of the 42 United Hospital Fund (UHF) neighborhoods in NYC and also to a zip code tabulation area based on their zip code at the time of report. UHF neighborhoods are ranked on an annual basis according to the case rate of early latent syphilis among females. Pregnant women were defined as living in a high morbidity neighborhood if they resided in a UHF neighborhood that ranked among the 10 neighborhoods with the highest early latent syphilis case rates among females (top 23.8% neighborhoods) in the year of their syphilis diagnosis.

§§ Neighborhood poverty categories were assigned in alignment with local area-based poverty guidelines. Pregnant women were defined as living in a high poverty neighborhood if they resided in a zip code tabulation area that was categorized as having high or very high poverty. https://www1.nyc.gov/assets/doh/downloads/pdf/epi/epiresearch-SES-measure.pdf.

¶¶ Data on health insurance coverage and prenatal care come from the Pregnancy Risk Assessment Monitoring System, an ongoing population-based survey of new mothers in NYC designed to monitor maternal experiences and behaviors before, during, and after pregnancy. These data are representative of NYC resident women who had a live birth in 2015.

*** Information on expanded health insurance options for pregnant women in New York. https://www1.nyc.gov/site/ochia/find-what-fits/pregnant.page.

††† Ensuring that electronic medical records support syphilis screening is included in CDC Call to Action: Let’s Work Together to Stem the Tide of Rising Syphilis in the United States. https://www.cdc.gov/std/syphilis/syphiliscalltoactionapril2017.pdf.

§§§ The rate of congenital syphilis in NYC in 2016 was 9.2 per 100,000 live births, which is less than the national congenital syphilis rate in 2016 of 16.2 per 100,000 live births. https://www1.nyc.gov/assets/doh/downloads/pdf/std/std-quarterlyreport2017-4.pdf.
